# Priority of SARS‐CoV‐2 test, trace, and isolation in Japan

**DOI:** 10.1002/jgf2.409

**Published:** 2020-12-04

**Authors:** Yasuharu Tokuda, Kenji Shibuya, Kazumasa Oguro

**Affiliations:** ^1^ Muribushi Okinawa Center for Teaching Hospitals Okinawa Japan; ^2^ Institute for Population Health King's College London London UK; ^3^ Faculty of Economics Hosei University Tama Tokyo Japan

## 1 LIMIT OF SYMPTOM‐BASED STRATEGY

The SARS‐CoV‐2 causes considerable transmission of infection from presymptomatic or asymptomatic people.^1^ Symptomatic people can isolate themselves immediately after the symptom onset, but there are many symptomless but infected persons who are now aware of risk at possible transmission to others without any intent. Thus, if family has an infected asymptomatic or presymptomatic person and elderly or high‐risk persons, the infection is easily transmitted within the family. Also, in a closed workplace or restaurant, the infection is transmitted to others. For this unique characteristic, the sole use of symptom‐based strategy breaks down.^2^ We need another strategy in addition to it.

Many European and American countries could not contain the infection, and they have had a tremendous number of deaths. Nonetheless, in countries of western pacific regions, many countries (other than Indonesia, the Philippines, and Japan) have successfully contained it.^3^ Successful countries such as China, New Zealand, or Taiwan have implemented large‐scale PCR tests followed by contact trace and supported isolation.

Polymerase Chain Reaction tests in Japan from February to May 2020 were limited to those with severe symptoms. Reasoning at this time was that testing all patients could cause healthcare collapse. But this reasoning was scientifically invalid, because early identification and isolation of infected persons are the most effective public health intervention in nonimmune infectious epidemics regardless of magnitude of symptoms. In May 2020, the prime minister of Japan confessed that Japan has not had enough capacity to conduct large‐scale PCR test. However, Japan has been reluctant in developing it for unknown reasons.^4^ Nevertheless, we provide here a strategy that should be employed by the national and local governments in Japan.

## 2 WISE USE OF TEST‐BASED STRATEGY

Where an infectious disease epidemic is ongoing, the value of Rt, which is the real‐time effective reproduction number, is important. This can be regarded as the real‐time mean number of secondary infections produced by one infected person. The epidemic spreads when it exceeds 1, but if it is kept less than 1, it can be suppressed. It is important to implement early public health intervention that reduces it to less than 1 in areas with the highest R. The most powerful intervention that can lower R is lockdown (a request to refrain from locking down because it is not legally possible in Japan). However, the collateral damages of refraining from going out and refraining from doing business from April to June were enormous. Thus, we should seek another way to lower Rt.

Even if it is not possible to isolate everyone with infection, isolation to make *R* < 1 is sufficient for containment. By pushing the quarantine effort to achieve *R* < 1, the infection can be suppressed. Our recommendation is as follows: To do this, use PCR test, trace, and isolation (TTI) more wisely. In epidemic areas, people with symptoms need to be tested rapidly so that appropriate contact trace and isolation can be performed if test positives. Close contacts should then undergo tests swiftly. When PCR test is used for the purpose of infection containment by test‐based strategy, unlike tests for diagnostic purpose among individual patients, PCR test that detects viruses present in saliva and pharynx is the clinical gold standard test, and thus, the concept of false negatives and false positives disappears.

False‐negative PCR test result is a concept for diagnostic test for COVID‐19 infection in an individual patient. Test for the purpose of preventing transmission aims to capture infectiousness or transmissibility, and it investigates real‐time viral shedding from saliva or nasopharyngeal fluid. Therefore, if we consider the PCR test as the clinical gold standard test for infectiousness or transmissibility and if sampling and test procedures are performed appropriately, the real‐time sensitivity is almost 100%.

False‐positive test result is extremely rare in real‐time reverse transcription polymerase chain reaction test sampling and procedure. Real‐world data based on large‐scale population testing have shown very high rate of specificity greater than at least 99.998. One should not describe false positive for a patient in recovery phase who test positive from biologically inactive viral RNA, and it should be described as positivity in recovery phase. Biological inactivity can only be proven by viral culture which is only available in special biohazard laboratories.

Currently in Japan, PCR test has been recommended only for symptomatic patients, their close contacts, and international travelers. Antigen test has been introduced for international travelers. Frequent use of antigen test combined with PCR test confirmation would also be another candidate strategy which has been implemented by Slovakia.^5^ However, global comparison indicates that Japan has the lower number of tests despite having the bigger epidemic in western pacific countries and it should be recommended to expand test indication for the following three groups.

## 3 PRIORITY GROUP

The first target is patients, elderly, and staff of hospitals and elderly care institutions to protect high‐risk people and elderly. These people should receive PCR test regularly. Although the fatality ratio of COVID‐19 is not so high in the total population, it is very high in the elderly and people with underlying diseases.

Elderly shielding strategy was implemented in the United Kingdom and Sweden, but it failed. Staff who acquire the virus outside institutions brought it into them. It is important to thoroughly discourage the elderly, institutions or healthcare workers, and household persons living with elderly people to go to hot spot areas. However, if the epidemic density is growing throughout the nearby area, infected persons always come out silently into hospitals and elderly care institutions.

In fact, some areas of Tokyo, Nagasaki, and several other prefectures are currently undergoing a major strategic shift, albeit inadequately. The activity of mayors and local leaders has made it possible for mild and asymptomatic people to actively undergo test. Many hospitals have already introduced PCR tests on all patients at the time of admission, and the indications for the tests are expanding to staff of hospitals and institutions.

## 4 SECOND PRIORITY GROUP

Polymerase Chain Reaction test should be done more thoroughly for even asymptomatic persons in local areas where the infection has spread. These include traveling people from epidemic areas at domestic airports or seaports.

Theoretically, Rt can be reduced by 50% by finding and isolating 50% of people who can transmit the infection. Likewise, local areas with high Rt would be effectively prioritized as next testing. The infection density in local areas could be reduced by repeating aggressive PCR tests combined with trace and isolation.

## 5 THIRD PRIORITY GROUP

This population is essential workers or special social professional since they are required to continue working even in pandemic time. In Japan, they are teachers, childcare specialists, store workers, delivery service workers, housekeeping persons, professional sumo wrestlers, professional sports players, musicians, and artists. Koshien baseball game (high school baseball tournament) has been canceled, but if professional athletes cancel the season, they might lose income and some professionals might quit jobs. People with high risk of severe COVID‐19, such as sumo wrestlers, should have frequent tests, and other professionals should have it about weekly or biweekly. The test frequency may need to be increased according to the risk of severe illness if infected.

## CONFLICT OF INTEREST

The authors have stated explicitly that there are no conflicts of interest in connection with this article.
